# Connexin43 Inhibition Prevents Human Vein Grafts Intimal Hyperplasia

**DOI:** 10.1371/journal.pone.0138847

**Published:** 2015-09-23

**Authors:** Alban Longchamp, Florent Allagnat, Florian Alonso, Christopher Kuppler, Céline Dubuis, Charles-Keith Ozaki, James R. Mitchell, Scott Berceli, Jean-Marc Corpataux, Sébastien Déglise, Jacques-Antoine Haefliger

**Affiliations:** 1 Department of Vascular Surgery, Centre Hospitalier Universitaire Vaudois, Laboratory of Experimental Medicine, Lausanne, Switzerland; 2 Department of Surgery, Brigham and Women’s Hospital, Harvard Medical School, Boston, Massachusetts, United States of America; 3 Malcom Randall Veterans Affairs Medical Center and the Division of Vascular and Endovascular Surgery, University of Florida College of Medicine, Gainesville, Florida, United States of America; 4 Department of Genetics and Complex Diseases, Harvard School of Public Health, Boston, Massachusetts, United States of America; National Institute of Health and Medical Research, FRANCE

## Abstract

Venous bypass grafts often fail following arterial implantation due to excessive smooth muscle cells (VSMC) proliferation and consequent intimal hyperplasia (IH). Intercellular communication mediated by Connexins (Cx) regulates differentiation, growth and proliferation in various cell types. Microarray analysis of vein grafts in a model of bilateral rabbit jugular vein graft revealed Cx43 as an early upregulated gene. Additional experiments conducted using an *ex-vivo* human saphenous veins perfusion system (EVPS) confirmed that Cx43 was rapidly increased in human veins subjected *ex-vivo* to arterial hemodynamics. Cx43 knock-down by RNA interference, or adenoviral-mediated overexpression, respectively inhibited or stimulated the proliferation of primary human VSMC *in vitro*. Furthermore, Cx blockade with carbenoxolone or the specific Cx43 inhibitory peptide ^43^gap26 prevented the burst in myointimal proliferation and IH formation in human saphenous veins. Our data demonstrated that Cx43 controls proliferation and the formation of IH after arterial engraftment.

## Introduction

Bypass of stenotic arteries with autologous saphenous vein is an established treatment for ischemic vascular disease. However its long-term success is limited, with 30–50% of the saphenous grafts failing within 5 years post implantation [1]. Vein graft failure is due to intimal hyperplasia (IH), a process whereby the vein wall thickens and expands to adapt to the high arterial pressure and flow.

Several studies suggest that intercellular signaling regulates VSMC’s phenotypic switch [2, 3] and proliferation [4–7]. However gap junction’s response to flow, pressure and their contribution to IH remains poorly delineated. The direct intercellular exchange of small molecule (<1kDa) through gap junctions is critical to integrate individual cells into coordinated multicellular units, allowing maintenance of cellular homeostasis and regulation of cellular functions including proliferation, migration and differentiation [8–10]. Endothelial cells (EC) predominantly express Cx37, Cx40 and some Cx43, whereas vascular smooth muscle cells (VSMC) are mostly coupled by Cx43 and Cx45, albeit minimal levels of Cx37 and Cx40 have been reported [8–10].

To determine the contribution of hemodynamic forces in the development of IH, we previously developed a model of bilateral rabbit carotid artery interposition grafts plus a unilateral distal outflow ligation to model differential flow states [11, 12]. In this setting, we identified divergent patterns of vein graft remodeling under low versus high flow and shear stress [12]. To further study the impact of hemodynamic forces after human vein arterial engraftment, we developed an *ex-vivo* human vein perfusion system (EVPS) [13, 14] and observed a 7-day high-pressure perfusion (mean = 100 mmHg) to be sufficient for IH formation. The VSMC forming the media layer of the vessels are highly specialized contractile cells featuring a very low proliferation rate under physiological conditions [15]. Following injury, VSMC integrate signals that promote their phenotypic switch from a quiescent contractile to a proliferative and motile state. The proliferation and migration of VSMC from the media toward the intimal layer are keys component to IH.

The aim of this study was to characterize the regulation of vascular connexins flowing arterial implantation of vein grafts. We observed that endothelial connexins Cx37 and Cx40 are rapidly down-regulated while VSMC connexin Cx43 is strongly and rapidly up-regulated in response to pulsatile arterial perfusion in rabbit vein grafts *in-vivo* and in human veins grafts *ex-vivo*. We further demonstrate, using primary human VSMC, that Cx43 controlled proliferation and show for the first time that specific Cx43 blockade partially prevented IH formation in human veins *ex-vivo*.

## Materials and Methods

### Animal vein graft

Male New Zealand White rabbits (3.0–3.5 kg; n = 56) were anesthetized through an intramuscular injection with ketamine hydrochloride (30.0 mg/kg). Anesthesia was maintained with endotracheal intubation and continuous isoflurane inhalant anesthesia (2% during painful stimuli, and 1% at latent periods). Vein bypass grafts were constructed with an anastomotic cuff technique as previously as described [11, 12]. Bilateral carotid artery interposition grafting with jugular vein and unilateral distal carotid artery branch ligation were performed to create two distinct flow states. Briefly, differential flow states between the right and left vein grafts were accomplished by ligation of the internal carotid artery and three of the four primary branches of the external carotid artery, resulting in an immediate six fold difference in mean shear stress [11, 12]. Routine daily postoperative care and analgesia was provided, including daily neurologic assessment up to day 5 post surgery to identify any or all of head tilt, ear droop, or extremity weakness. Graft flow rates were determined at the time of implantation and harvest with an ultrasonic flow meter (VisualSonics Inc, Toronto, Ontario, Canada). Vein grafts harvest was performed under general anesthesia through an intramuscular injection with ketamine hydrochloride (30.0 mg/kg). Then the recipient rabbit was whole-body blood flushed with saline via intravascular access through the left ventricle, leading to exsanguination and death. Vein grafts were harvested after implantation at 2 hours (0.08 days) (high-flow, n = 4; low-flow, n = 4) and 1 day (high-flow, n = 5; low-flow, n = 4), 3 days (high-flow, n = 4; low-flow, n = 5), 7 days (high-flow, n = 5; low-flow, n = 5), 14 days (high-flow, n = 4; low-flow, n = 5), and 28 days (high-flow, n = 6; low-flow, n = 6). Normal rabbit jugular vein served as the control (n = 5). The perianastomotic regions for each vein graft were removed, and the mid-portion of the graft was divided into two segments, one for histological and immunohistochemical analyses and the other for total RNA extraction using the RNeasyMiniKit (Qiagen) as previously described [12].

### Microarray analysis

Microarray analysis was performed as previously described [12]. Briefly Complementary DNA form rabbit vein was generated using Ovation Pico WT kit (NuGEN, San Carlos, Calif) and labeled using GeneChip WT Terminal Labeling (Affymetrix, Santa Clara, Calif). Samples were hybridized to a proprietary rabbit array (Affymetrix). The resulting expression data were normalized with the Partek Genomics Suite (Partek, St. Louis, Mo).

### Human saphenous veins culture

After informed consents, 27 surplus segments of non-varicose human saphenous veins were obtained from 27 patients (15 men and 12 women) with a median age of 72 years (interquartile range 51–82), who underwent lower limb bypass surgery for critical ischemia. A 7 to 9 cm long segment of the greater saphenous vein, with an external diameter of 2.5–4 mm, was harvested and immediately stored at 4°C in a RPMI-1640 Glutamax medium, supplemented with 12.5% fetal calf serum (Life Technologies Europe B.V.). The segment was divided in 3 equal parts. One part was immediately fixed in either formalin (one half) or rapidly frozen in liquid nitrogen (the other half). The two others parts from the same vein were perfused using an *ex-vivo* perfusion system (EVPS) [13, 14, 16], with or without an external mesh reinforcement (ProVena, B.Braun Medical SA) to model and control (mesh) the development of IH as previously described [13] (more details on the method can be found at: http://www.jove.com/video/52079/procedure-for-human-saphenous-veins-ex-vivo-perfusion-external). A pulsatile, cardioid signal at 60 pulses per minute with constant amplitude was setup up via the computer software which independently pilots the gearing pump. The resulting flow was about 160 milliliters per minute. The conditions of the perfusion were set to obtain a shear stress (SS) of 9–15 dyn/cm^2^, as expected in the femoral artery [14], given by SS = 4 μQ/ π r^3^, where μ is the viscosity of the perfusion medium set to was 3.73 10^−2^ dyn·s/cm^2^, as measured in a Coulter viscometer (Coulter Electronics, High Wycombe, UK), Q the flow rate (mL/s), and r the radius (cm) of the vein segment. Mean pressure (MP) = 100 mmHg, as given by MP = (systolic pressure + 2 x diastolic pressure)/3. Upon completion of the perfusion period, the vein segments were dismounted and the 5 mm proximal and distal ends, which attached the vessel to the perfusion equipment, were discarded.

Static vein cultures were performed as previously described [17]. Briefly, 5 mm segments of vein were opened longitudinally and pinned on a layer of Sylgard 184 resin (Dow Corning, Seneffe, Belgium) in a Pyrex dish and kept in culture for 10 days in RPMI-1640 Glutamax supplemented with 10% FBS and and 1% antibiotic solution (10,000 U/mL penicillin G, 10,000 U/mL streptomycin sulphate). 100 μM Carbenoxolone (Sigma-Aldrich), 200 μM of the Cx-mimetic peptides ^43^Gap26 (VCYDKSFPISHVR, Tocris Bioscience Bristol, UK) and the control scrambled peptide (PSFDSRHCIVKYV) (Severn Biotech, UK) were prepared in SMC culture media. 5-mm segments of vein were harvested after culture. The vein segments were either frozen for molecular analysis or fixed in 4% formalin and paraffin-embedded for histological analysis.

### Morphometry

Hematoxylin-eosin and Van Gieson-elastin (VGEL) stainings were used for histological and morphometric analysis, respectively. All morphometric measurements were done by 2 independent researchers, one of them blind to the experimental groups using the Leica Qwin**®** software (Leica, Switzerland). Twenty-four measurements of the thickness of the intima and media layers were made in each sample at a magnification of x100 as previously described [14, 16].

### Cell culture

The human smooth muscle cells were prepared from explants culture, as previously described [18–20]. Briefly, primary smooth muscle cells were cultured from human saphenous veins from a similar cohort used for *ex-vivo* perfusion. Veins explants of 1–2 mm were plated, luminal side down, on the dry surface of a 24-well culture plate, previously coated with 1.5% Gelatin type B (Sigma-Aldrich). Explants were gently covered with one drop of RPMI, 10%FBS medium, and placed overnight in a 37°C, 5% CO2. The next day, culture medium was carefully added to the wells, taking care not to detach the explants. VSMC were identified by immunostaining using antibodies to smooth muscle actin (abcam, ab5694) and desmin (Dako, M 0760). Passages 1 to 4 were used for the experiments.

### RNA interference

Smooth muscle cells were transiently transfected using lipofectamine RNAiMAX (Life Technologies Europe B.V.) and 30nM siRNA. Cx43 siRNAs 1 and 2 (s5758 and s5759) were purchased from Ambion (Ambion, Life Technologies Europe B.V., Zug, Switzerland). Briefly, confluent cells were transfected using antibiotics free Optimem medium (Life Technologies Europe B.V.), according to the manufacturer’s indications. Cells were kept 48 hours in culture before the experiments. The Allstars Negative Control siRNA (Qiagen, Hombrechtikon, Switzerland) was used as a control. The efficiency of transfection was above 90% as evaluated using the Thermo Scientific™ Dharmacon™ siGLO™ Green Transfection Indicator (Thermo Fisher Scientific Inc. Waltham, MA, USA).

### Adenoviral transfer

The Control Ad-GFP virus were generated as previously described [21, 22]. Ad-Cx43 was a kind gift from Professor Viviana Berthoud and Eric Bayer (University of Chicago, Illinois, U.S.A.). All viral vectors were amplified and purified by Vector Biolabs, (Philadelphia, PA, U.S.A.). Human VSMC were infected overnight in complete medium and collected 48 hours later.

### Quantitative real-time PCR

Quantitative real-time PCR analysis was performed on total RNA extracted from human saphenous veins using the TriPure isolation reagent (Roche) as previously described [14, 20]. The primers used to amplify specific cDNAs are given in S1 Table, and were designed using the free online software Primer3Plus (http://primer3plus.com/cgi-bin/dev/primer3plus.cgi) [23]. Levels of expression were determined relative to those of GAPDH, which were not significantly altered by the experimental conditions tested here.

### Western Blots

Segments of saphenous veins were reduced to powder in liquid nitrogen, and homogenized in lysis buffer as published [23, 24]. Immunoblot analyses were performed as previously described [16, 23, 25] using the following antibodies: rabbit polyclonal against Cx43 (Millipore) diluted 1:1000; rabbit polyclonal against Cx40 (InVitrogen) diluted 1:2000; rabbit polyclonal against Cx37 (kindly provided by Pr. A. Simon [23]) diluted 1:2500; mouse monoclonal against α-Tubulin (Sigma-Aldrich), diluted 1:1000 and horseradish peroxidase-conjugated goat anti-rabbit or anti-mouse IgG (Sigma-Aldrich), diluted 1: 5000.

### Immunohistochemistry and immunocytochemistry

The Proliferating Cell Nuclear Antigen (PCNA) and alpha smooth muscle actin (α-SMA) immunostaining were performed on paraffin-embedded tissue slides using the monoclonal mouse anti-human PCNA (Clone PC10 1/200; Dako Schweiz AG, Baar, Switzerland), and anti-SMA (Abcam, ab5697, Lucerna chem. AG, Luzern, Switzerland) antibodies. Slides were subjected to standard protocol of antigen retrieval prior to antibody incubation. PCNA staining was revealed either using DAKO envision^TM^+-HRP-DAB staining or using secondary biotin-steptavidin antibodies coupled to AlexaFluor 594. The PCNA positive signal was evaluated automatically using the ImageJ software on 10 slides per vein and 3 to 5 veins per conditions [16]. PCNA/DAB^+^ signal were quantified as follows: images were converted into 16 bit and a color threshholding based on red hue was applied to select only the brown staining. The selected staining was processed into a binary image and the number of positive pixels determined. This signal was normalized to the total pixel number composing the vein section and expressed as a PCNA positive pixels/total vein pixels ratio. PCNA fluorescent signal were quantified as follows: images were converted to a 32 bit format and the signal to noise ratio was determined by applying the Yen thresholding method. A binary image was then created and the number PCNA^+^ nuclei automatically detected. Data were normalized to the number of cells (nuclei imaged by DAPI staining) in each image. Cx immunostainings were performed using frozen sections of unfixed veins as previously described [23], using the primary antibodies used for Western blotting at a dilution of 1:100, except those against Connexin43 (Millipore), which was diluted 1:200. Vein sections were rinsed in PBS, counterstained with 0,02% Evans Blue and coverslipped. BrdU and Cx43 immunostaining were performed as previously described [20, 26] on SMC grown on glass coverslips and fixed for 5 min in −20°C acetone, air-dried, rinsed in PBS and permeabilized for 1 h in PBS supplemented with 2% BSA and 0.1% Triton X-100 (full PBS). Brdu positive nuclei were automatically detected using the ImageJ software and normalized to the total number of DAPI-positive nuclei.

### Cell cycle analysis

To measure the cellular DNA content, transfected cells were kept under 0% FBS for 48 hours, and then supplemented with 10 ng/mL Platelet-Derived Growth Factor (PDGF-BB, PeproTech) for an additional 24 hours. Adherent cells were collected by trypsinization. Cells were pelleted, rand washed in PBS/EDTA and fixed in 70% ethanol overnight at 4°C. Cells were pelleted and resuspended 15 min at 37° in 1ml PBS 0.1% Triton X-100 with 0.1 mg RNase (Sigma-Aldrich) and 0.2 mg propidium iodide as previously described [27]. Samples were analyzed on BD FACS Scan. The percentage of cells in different phases was calculated using FlowJo software (Tree Star, Inc).

### Scratch wound assay

Two perpendicular scratch wounds were created on confluent cells using a sterile p200 pipette tip. Repopulation of the wounded areas was recorded by phase-contrast microscopy connected to a digital camera every 2 hours and for 24 hours. The size of the denuded area was determined at each time point from digital images using the ImageJ software. The area of each experiment, measured immediately after the wounding was used to standardize quantitative analysis.

### Statistical Analysis

All experiments were quantitatively analyzed and results shown as mean + SEM. One or two-way analysis of variance (ANOVA) were performed to compare mean values between groups, using post hoc t-tests with Bonferroni correction, as provided by the Statistical Package for the Social Sciences (SPSS 17.0, Chicago, Ill., USA). Statistical significance was set at p < 0.05.

### Ethics Statement

Written, informed consent was obtained from all vein donors who underwent lower limb bypass surgery for critical ischemia. The study protocols for organ collection and use were reviewed and approved by the Centre Hospitalier Universitaire Vaudois (CHUV) and the Cantonal Human Research Ethics Committee (http://www.cer-vd.ch/, no IRB number, Protocol Number 170/02), and are in accordance with the principles outlined in the Declaration of Helsinki of 1975, as revised in 1983 for the use of human tissues.

Rabbit care, surgery and euthanasia procedures were approved by the Institutional Animal Care and Use Committee of the University of Florida (https://iacuc.ufl.edu) and conforms to the Guide for the Care and Use of Laboratory Animals (NIH Publication, Revised 2011, Animal Welfare Assurance Number A3377-01).

## Results

### Arterial engraftment induces vein intimal hyperplasia and time-dependent regulation of the vascular connexins

**Fig 1:**
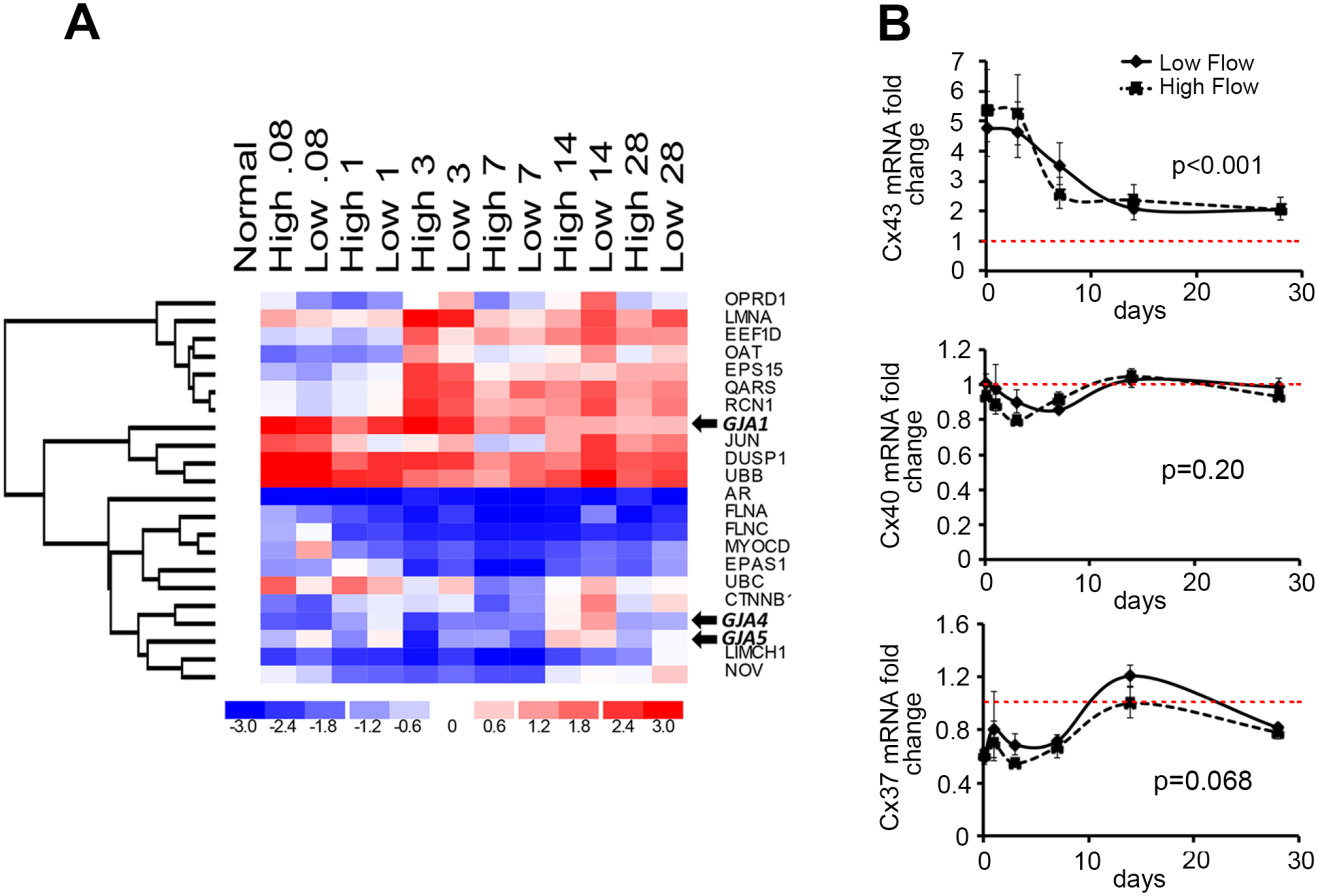
Probe sets analysis demonstrate changes in Cx transcripts levels over time after rabbit vein graft. **A)** Supervised hierarchal clustering analysis of selected time-dependent probe sets (false discovery rate = .05) reveals GJA1 (Cx43), GJA4 (Cx37) and GJA5 (Cx40) to cluster with differential expression patterns. Mean expression data at each time point (expressed as day) and flow condition are presented across the x-axis, with red representing upregulation and blue representing downregulation compared to normal vein. **B)** Mean expression fold change from baseline (normal vein) of Cx43 (**top**), Cx40 (**middle**) and Cx37 (**bottom**) at each time point under high and low flow conditions. Dotted red line represents the mean value observed in control normal vein. Data represent mean ± SEM of 5 experiments.

As previously described [11, 12, 28], bilateral rabbit vein grafting caused a robust hyperplastic response beginning at 7 days after vein graft implantation, and progressing to 28 days. Microarray analysis of both vein grafts demonstrated that flow did not impact the gene expression of Cx37 (GJA4), Cx40 (GJA5) and 43 (GJA1), as assessed by ANOVA for fixed effects with flow and time as the two variables (Fig 1 and S1 Fig). Temporal changes in gene expression after vein graft placement were then analyzed from 2 hours (0.08 days) following grafting and up to 28 days. At a *p* value < 0.05, 57 genes were differentially expressed over time (S1 Fig). Unsupervised genes cluster analysis revealed that individual vein grafts cluster by time point rather than by flow state, indicating time from implantation as the dominant effect on gene expression (S1A Fig). A supervised hierarchal clustering analysis using averaged expression data further revealed that among the early upregulated genes (S1B Fig), Cx43 was the most differentially expressed, with a fivefold upregulation 2 hours after implantation, which persisted over time (Fig 1A and 1B). In the cluster of downregulated genes, despite an early down-regulation, the Cx37 and Cx40 transcript levels did not significantly change over time (Fig 1A and 1B).

### Myointimal proliferation and Cx43 are increased in human veins exposed to arterial hemodynamic

**Fig 2:**
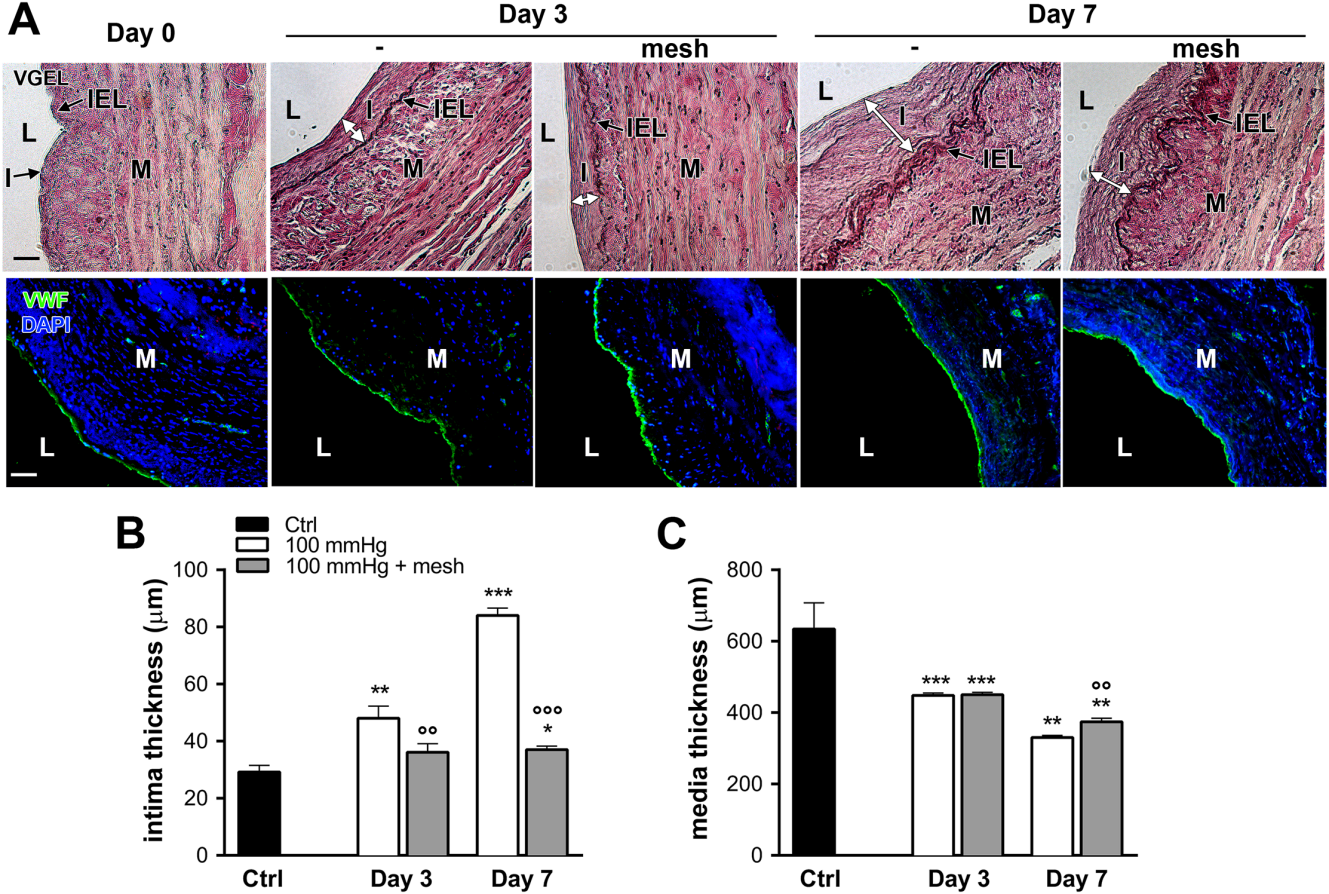
Intimal hyperplasia formation in *ex-vivo* human vein perfusion. Human veins were subjected to arterial pressure and flow (mean pressure = 100, high flow) for 3 and 7 days using an *ex-vivo* perfusion model. **A)** Representative Van Gieson Elastic Laminae staining (VGEL, **upper panel**) and vWf (green) and nuclei (DAPI blue) (**lower panel**) staining. I: intimal hyperplasia; L: lumen; M: media; IEL: internal elastic lamina. Bar represents 100 μm. Data are representative of 8–9 experiments. **B-C)** Morphometric measurements of human veins subjected to a 3- and 7-day pulsatile high pressure perfusion, with or without external mesh reinforcement. Data represent mean ± SEM of 4–9 experiments. *p<0.05, **p<0.01 and ***p<0.001 versus the non-perfused veins (ctrl). °°p<0.01 and °°°p<0.001 versus the unsupported vein segment.

Using our validated *ex-vivo* vein perfusion model [13, 16], human saphenous veins grafts were exposed to high-pressure (mean = 100mmHg) and high flow (180-200ml/min), mimicking the femoral artery hemodynamic. As previously established [16], 3 and 7 days of arterial perfusion resulted in neointima formation (Fig 2A upper panel and 2B) and reduced media thickness (Fig 2A upper panel and 2C). Von Willebrand factor (vWF) immunostaining revealed intact endothelial cells lining the lumen of native vein and vein subjected to a 3 or 7 days high pressure pulsatile perfusion (Fig 2A, **lower panel**).

**Fig 3:**
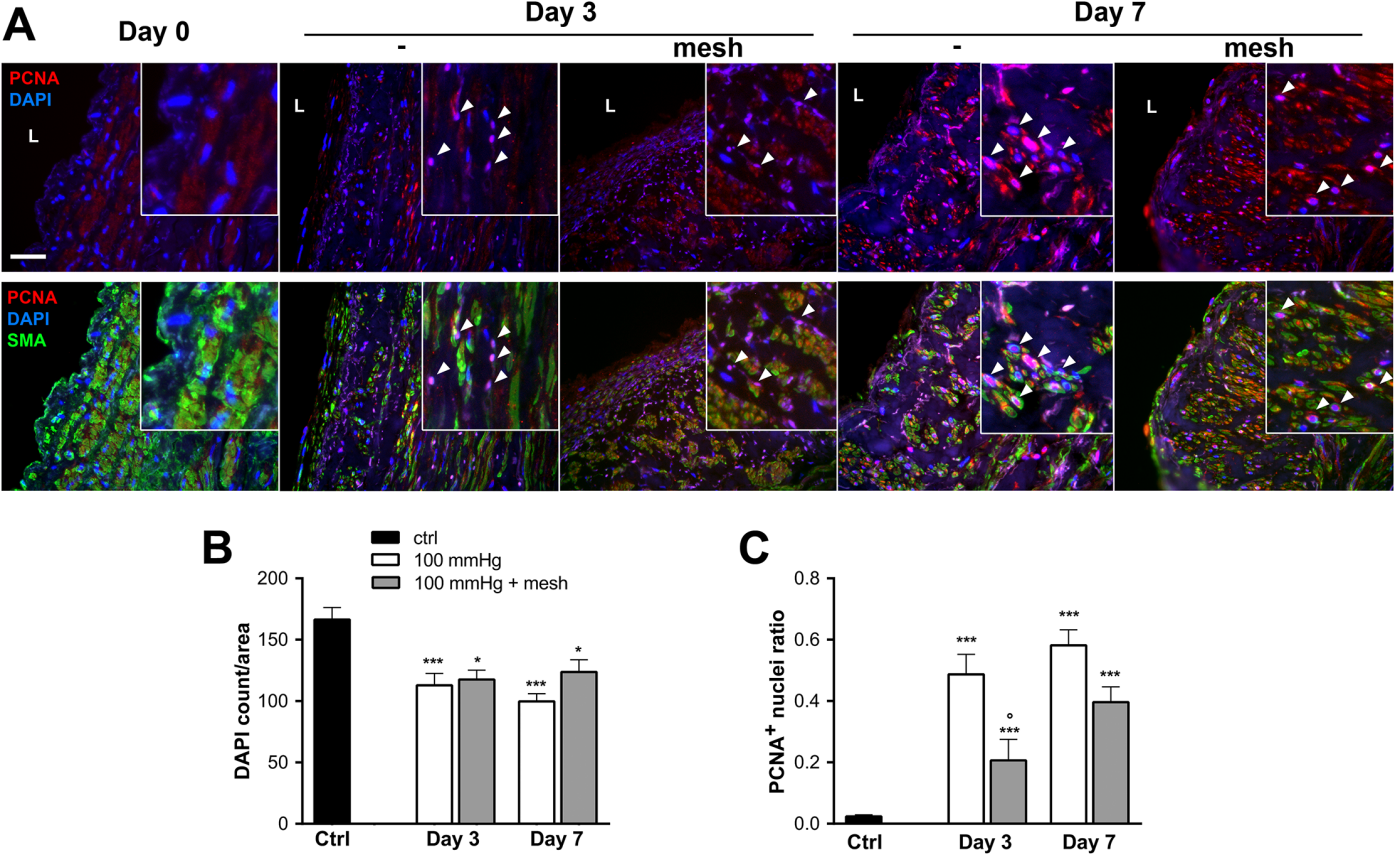
Growth dynamics in *ex-vivo* human vein perfusion. Human veins were subjected to arterial pressure and flow (mean pressure = 100, high flow) for 3 and 7 days using an *ex-vivo* perfusion model. **A)** Representative PCNA (red) and DAPI (blue) and smooth muscle actin (SMA; green). Arrowheads point to PCNA positive nuclei. L: lumen, Bar represents 50 μm. Data are representative of 8–9 experiments. Square insets represent a 4 fold magnification of images. Quantitative assessment of nuclei (DAPI) over section area (**B**) and PCNA positive nuclei over total DAPI positive nuclei (**C**). Data represent mean ± SEM of 4–9 experiments. *p<0.05 and ***p<0.001 versus the non-perfused veins (ctrl). °p<0.05 versus the unsupported vein segment.

Further analysis of cell growth dynamics, showed reduced media cellularity and a concomitant increase in proliferative activity within the myointima after 3 and 7 days (Fig 3), as assessed by PCNA immunostaining. The addition of an external reinforcement preserved the structure but not the cellularity of the media layer, reduced the number of PCNA-positive nuclei, and prevented the development of IH (Fig 3).

**Fig 4:**
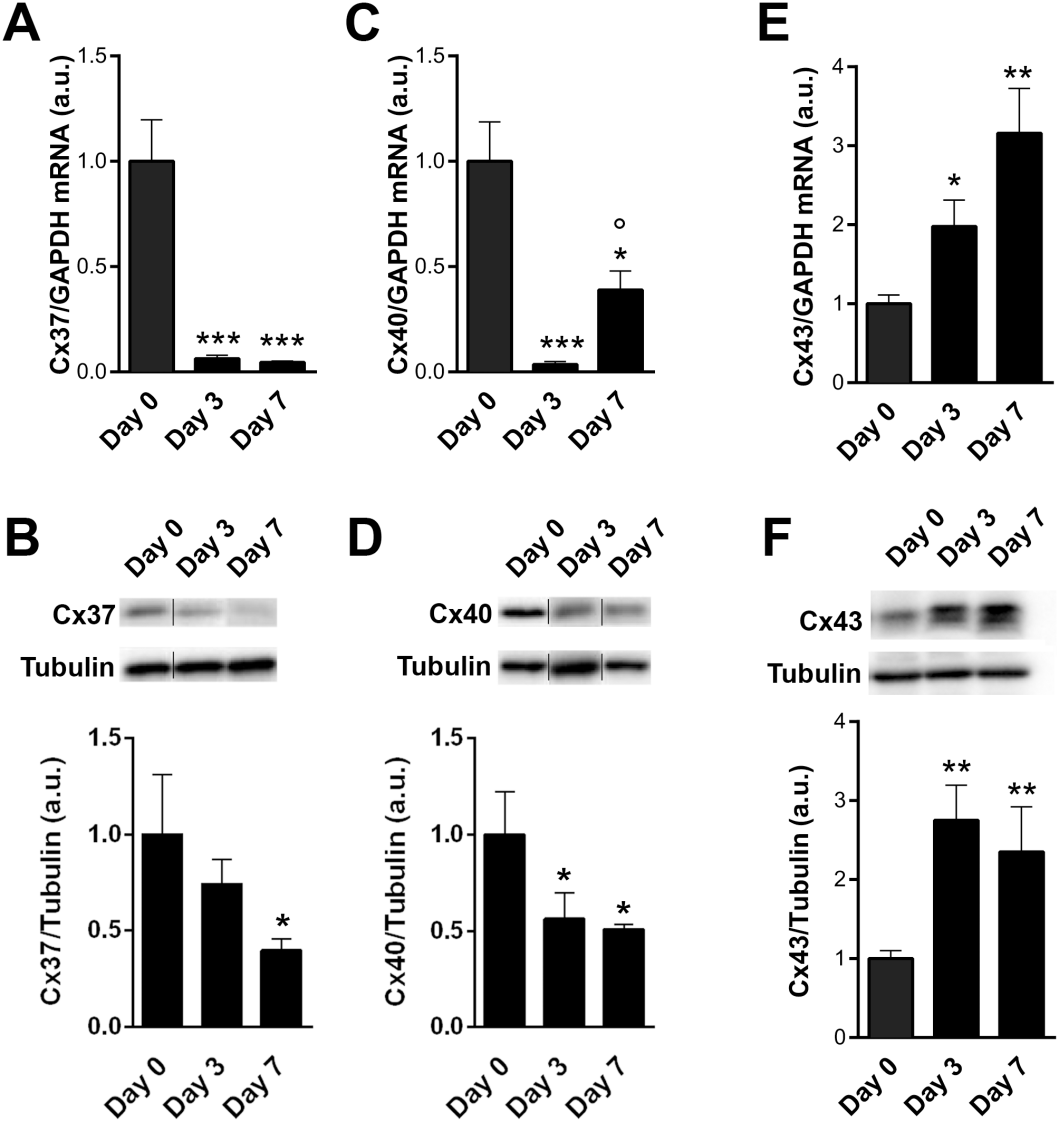
Expression of endothelial Cx37 and 40 are decreased, while muscular Cx43 is increased following arterial engraftment of human vein. Human veins were subjected to arterial pressure and flow for 3 and 7 days using an *ex-vivo* perfusion model. Levels of Cx37 transcript (**A**) and protein (B), Cx40 transcript (**C**) and protein (D), and Cx43 transcript (**E**) and protein (**F**). Data are mean ± SEM of 4–8 experiments. *p<0.05, **p<0.01 and ***p<0.001 versus the native segment. °p<0.05 day 3 versus day 7.

In human saphenous vein, Cx37 and Cx40 are specifically expressed in ECs, whereas Cx43 is mainly expressed in VSMC [17, 29]. Both Cx37 and Cx40 transcripts (Fig 4A and 4C) and protein (Fig 4B and 4D) levels decreased after 3 and 7 days of arterial hemodynamic exposure. In contrast, Cx43 mRNA (Fig 4E) and protein (Fig 4F) levels were both increased after 3 and 7 days.

**Fig 5:**
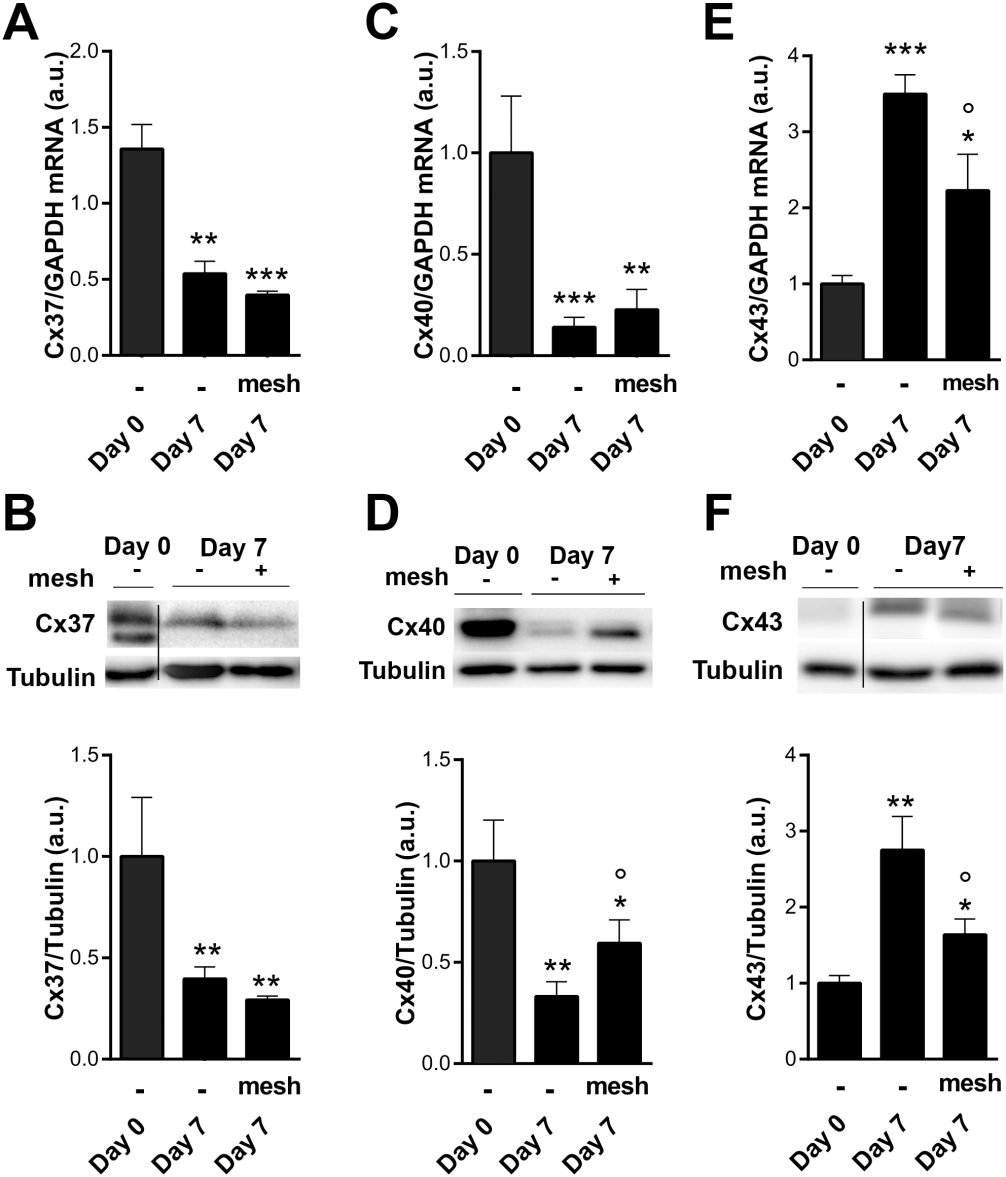
Cx43 overexpression following arterial engraftment is dampened by the use of an external mesh support. Human veins were subjected to arterial pressure and flow for 7 days in absence or presence of the external mesh support. Cx37 transcripts (**A**) and protein (**B**) levels remain low in presence of the mesh. Cx40 transcript (**C**) and protein (**D**) levels tend to increase in presence of the mesh. Cx43 transcript (**E**) and protein (**F**) levels are decreased by the external support. Data are mean ± SEM of 4–8 experiments. *p<0.05, **p<0.01, ***p<0.001 versus the native segment. °p<0.05 ctrl versus external mesh.

External vein support did not prevent Cx37 mRNA (Fig 5A) and protein decrease (Fig 5B). The external mesh had a tendency to reverse Cx40 mRNA (Fig 5B) and significantly prevented Cx40 protein drop (Fig 5D). In contrast, the mesh support dampened Cx43 mRNA (Fig 5E) and protein (Fig 5F) upregulation.

**Fig 6:**
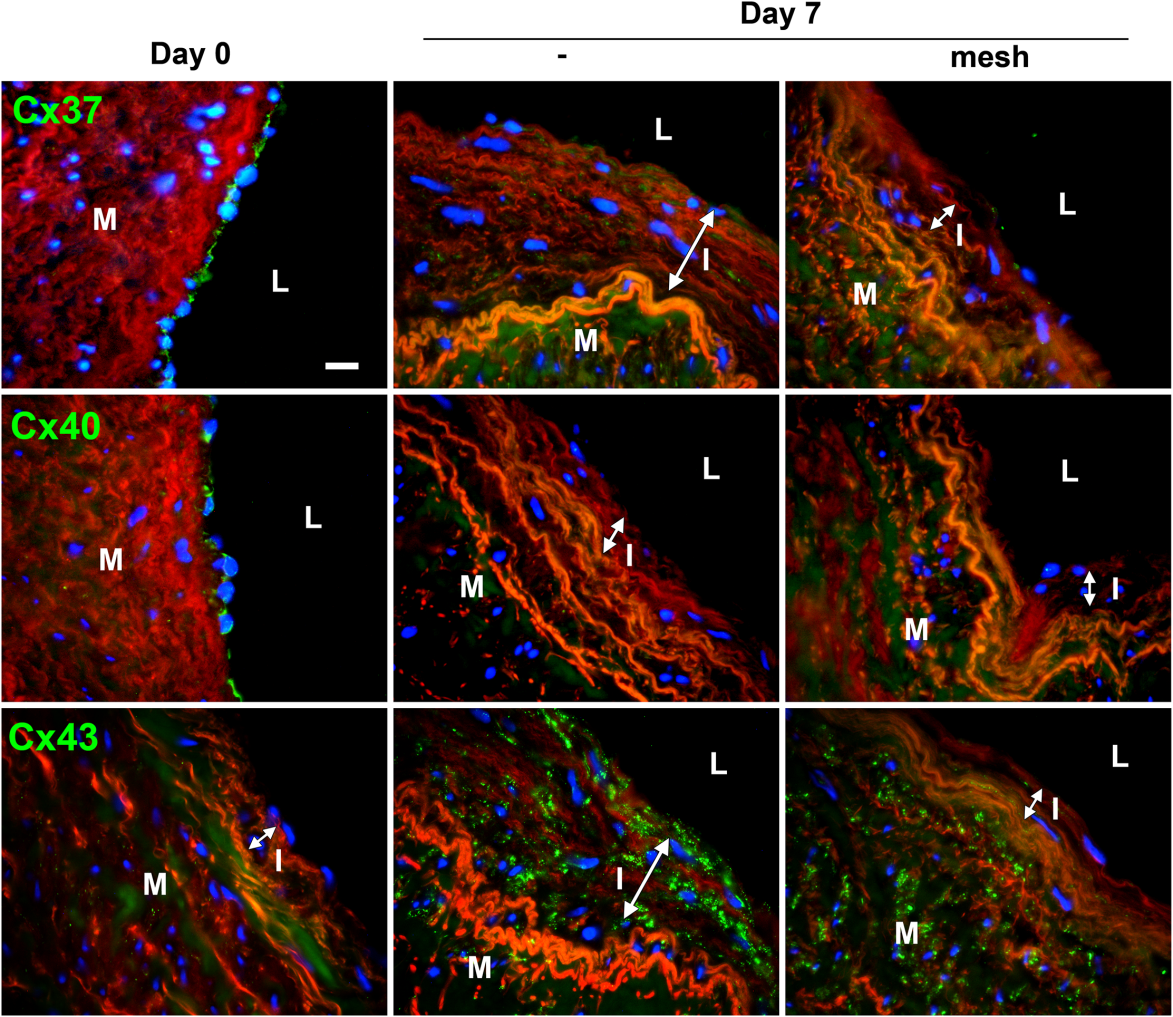
Expression pattern of endothelial Cx37 and 40 and muscular Cx43 following arterial engraftment of human vein. Human veins were subjected to arterial pressure and flow for 3 and 7 days using an *ex-vivo* perfusion model. Representative Cx37 (**upper panel**), Cx40 (**middle panel**) and Cx43 (**lower panel**) immunostaining after 7 days of perfusion, with or without external mesh. Connexin stainings (green) were counterstained with Evans blue staining of the elastic laminae (red). Data represent mean ± SEM of 8–9 experiments. Bar represents 50 μm. L: lumen; I: intimal hyperplasia; M: media. Data are representative of 5 experiments. Square insets represent a 3 fold magnification of images.

Further immunofluorescent staining of connexins after 7 days of perfusion confirmed that endothelial Cx37 and 40 remained decreased in presence of the mesh, whereas Cx43 overexpression was attenuated (Fig 6).

### Cx43 controls smooth muscle cell proliferation

**Fig 7:**
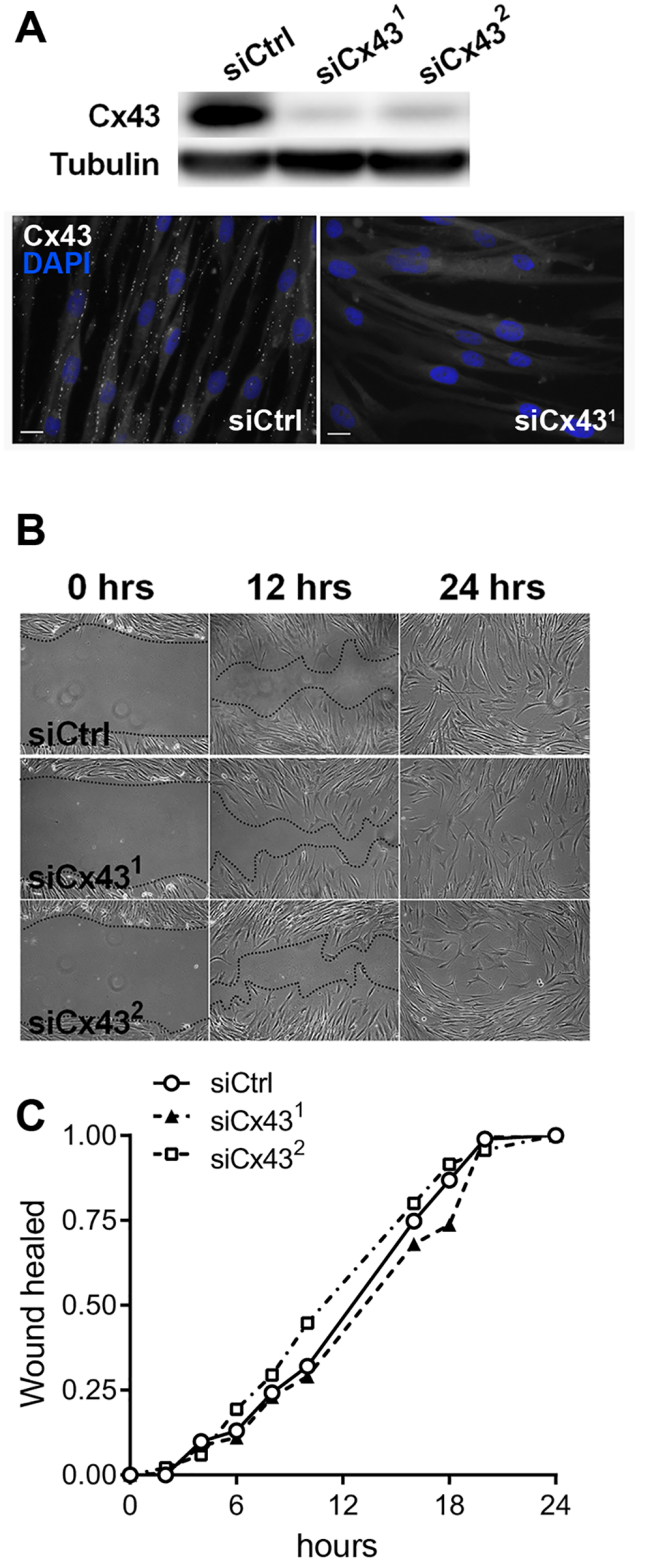
Cx43 knock-down reduces hVSMC proliferation. Human primary VSMC were transfected with a control siRNA (siCtrl) or Cx43 siRNAs (siCx43^1^ and siCx43^2^). **A)** Representative Western blot analysis of the Cx43 over tubulin levels (**upper panel**, n = 3) and immunofluorescence staining (**lower panel**, n = 3). **B)** Human VSMC migration was assessed using scratch wound assay. Representative images of wound closure at 0, 12 and 24 hours after injury of VSMC transfected with a control siRNA (siCtrl) or two different Cx43 siRNAs (siCx43^1^ and siCx43^2^). **C)** Representative wound closure kinetic (n = 3–4) in cells transfected with the siCtrl (continuous line), siCx43^1^ (dotted line) or siCx43^2^ (dashed line).

**Fig 8:**
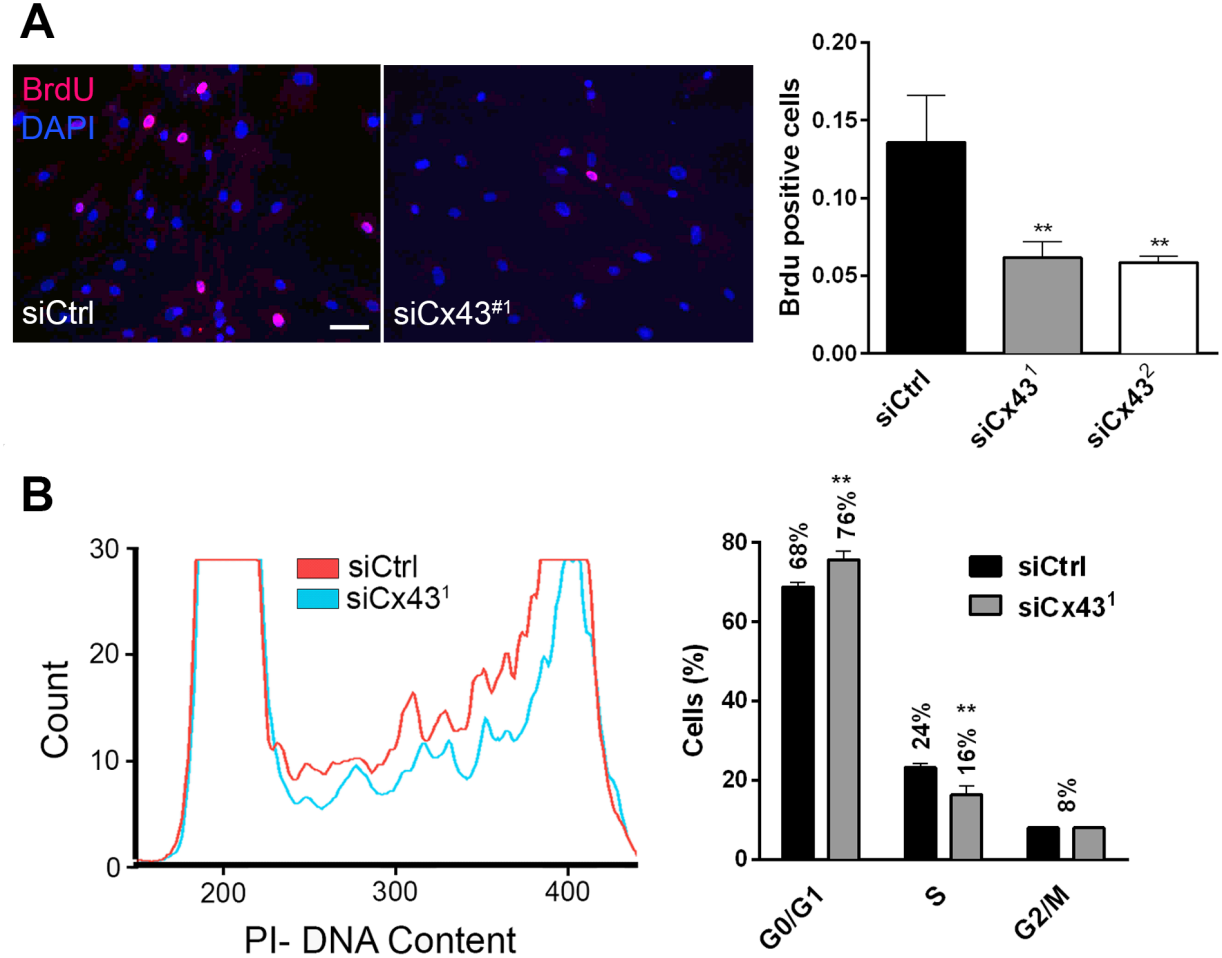
Cx43 knock-down does not affect human VSMC migration. Primary hVSMC were treated 24h with PDGF-BB, 48 hours after transfection with a control (siCtrl) or Cx43 (siCx43^1 or 2^) siRNAs. **A**) Representative immunofluorescence staining (left panel) and quantification of Brdu positive nuclei (right panel). Nuclei were identified with DAPI. Data represent mean ±SEM of 5 experiments. **p<0.01 versus siCtrl. Bar represents 20 μm. **B**) Representative (left panel) and quantification (right panel) of flow cytometry analysis of DNA content (propidium iodide staining). Data represent mean ±SEM of 4 experiments. **p<0.01 versus siCtrl.

**Fig 9:**
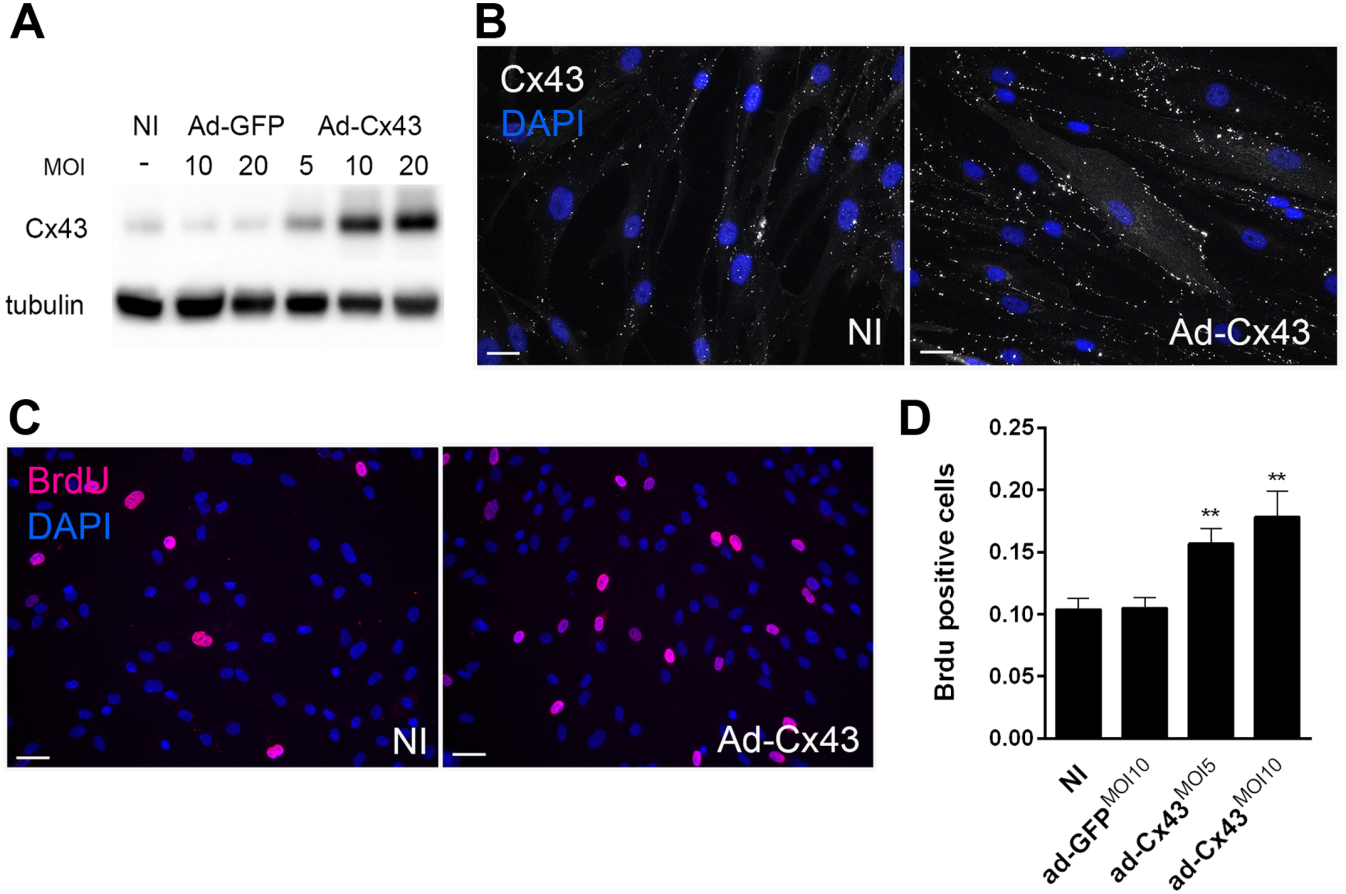
Cx43 overexpression promotes SMC proliferation. hVSMC were infected or not (NI) with a control adenovirus (Ad-GFP) or a human Cx43 adenovirus (Ad-Cx43) at various multiplicity of infection (MOI) as indicated. **A and B)** Representative Western blot analysis of the Cx43 over tubulin levels and Cx43 immunofluorescent staining. **C and D)** Representative immunofluorescent images and quantification of Brdu incorporation during 24h in primary hVSMC in presence of PDGF-BB. Nuclei were identified with DAPI. **B**) Bar represent 50 μm. **C**) Bar represent 25 μm. Data represent mean ±SEM of 5–6 experiments. **p<0.01 versus NI and ad-GFP.

To determine the role of Cx43 in VSMC phenotypic switch, Cx43 was knocked-down in primary human VSMC using two distinct siRNAs, which both decreased Cx43 protein levels by 90% (Fig 7A). Loss of Cx43 (siCx43^1 and 2^) did not alter VSMC migration, as compared to VSMC transfected with a control siRNA (siCtrl) (Fig 7B and 7C). However, the absence of Cx43 reduced by 40% the number of Brdu positive cells over a 24h treatment with 10 ng/mL PDGF-BB (Fig 8A). Flow cytometry analysis of DNA content further revealed that the absence of Cx43 arrested VSMC in G0/G1 cell cycle phase (76% vs 68%) (Fig 8B). Conversely, the adenoviral-mediated overexpression of human Cx43, which resulted in a dose-dependent increase in Cx43 protein levels (Fig 9A) at the cell-cell membrane contacts (Fig 9B), increased VSMC proliferation by 30% over a period of 24 hours in presence of 10 ng/mL PDGF-BB (Fig 9C and 9D).

### Connexins blockade prevents intimal hyperplasia

**Fig 10:**
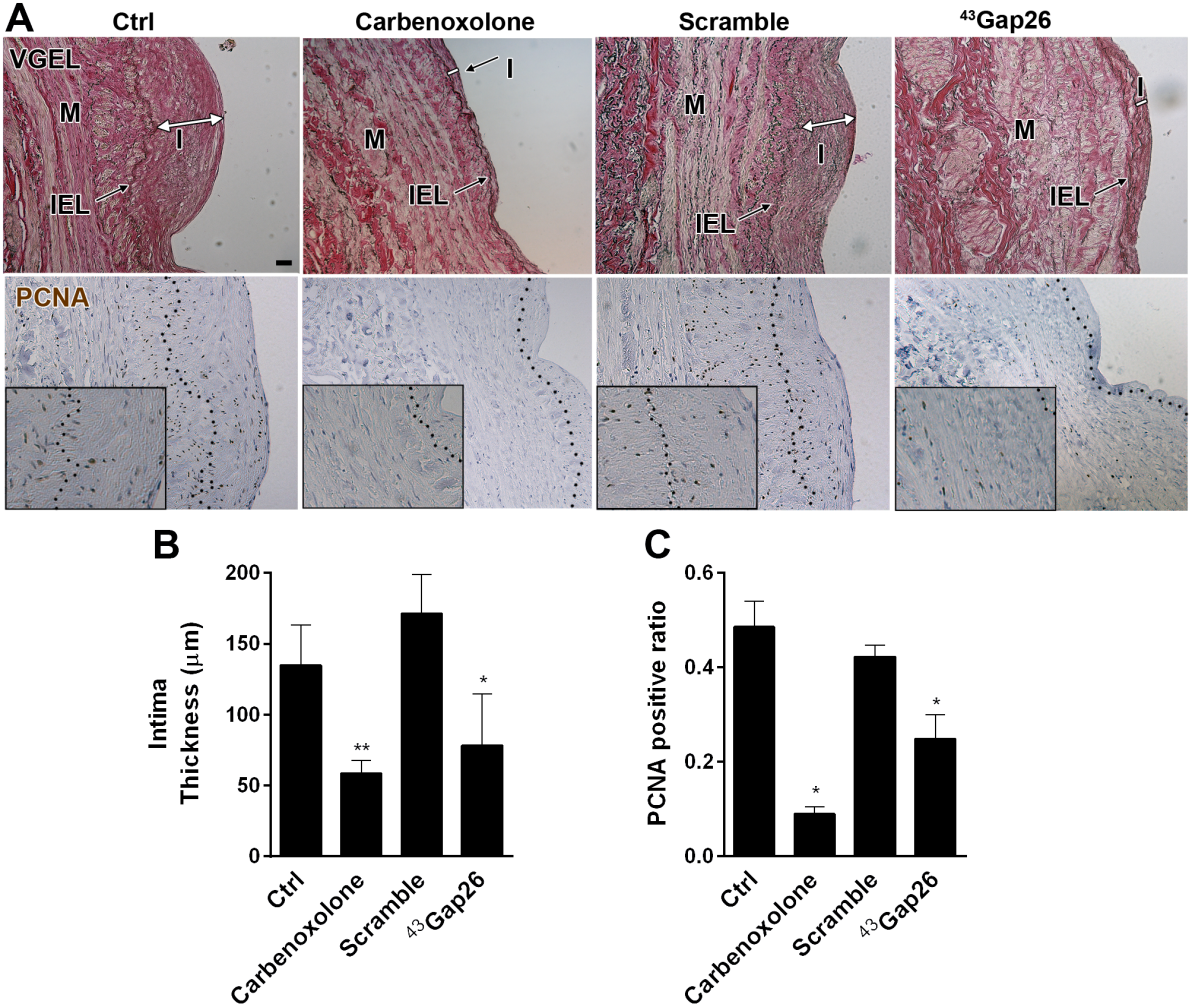
Inhibition of Cx43-mediated intercellular communication alleviates the development of intimal hyperplasia in human veins. 5 mm segments of opened vein were kept in static culture for 10 days in presence or not (**ctrl**), of 200 μM carbenoxolone or 200 μM ^43^Gap26 peptide or a respective scramble peptide. **A)** Representative VGEL (**upper panel**), and PCNA (**lower panel**) stained sections showed decreased intimal thickness and cell proliferation in presence of the Cx43 inhibitors. I = intima; M = media; IEL: internal elastic lamina. Bar represents 100 μm. Square insets in PCNA staining represent a 3 fold magnification of images. **B and C)** Quantitative assessment of intima thickness (**B**) and PCNA positive area (**C**). Data represent mean ± SEM of 3–4 experiments. *p<0.05, versus ctrl.

The potential vasculoprotective effect of Cx43 blockade was then tested in human saphenous vein, using the pan-Cx inhibitor carbenoxolone (100 μM) or the specific Cx43 inhibiting peptide ^43^Gap26 (200 μM) [30, 31]. Both strategies reduced the development of IH (Fig 10) and myointimal proliferation (PCNA staining) (Fig 10) observed after 10 days of static culture, suggesting that Cx43 is the main Cx involved in VSMC proliferation and IH.

## Discussion

The data here show that arterial engraftment rapidly increased rabbit Cx43 gene expression *in-vivo*. Consistently, Cx43 transcript and protein levels were upregulated in human veins exposed to arterial hemodynamics *ex-vivo*. Cx43 genetic inactivation or chemical inhibition reduced VSMC proliferation in vitro and in vivo, reducing myointimal proliferation and IH formation.

Cx43 was rapidly and consistently increased in all our experimental models, which supports findings from previous studies showing higher Cx43 levels in vascular occlusive disorders, including rabbit iliac and rat carotid restenosis models [32, 33], or atherosclerotic lesion in rabbit aorta [34, 35]. We further observed that flow, which play a key role in intimal expansion and VSMC proliferation [12, 28], is not involved in Cx43 overexpression, in accordance with our previous findings that Cx43 expression is increased in static human vein culture [17]. The fact that Cx43 is upregulated both in vivo and ex-vivo implicate intrinsic factors in the Cx43 upregulation following vein grafting. Similarly, the contribution of bone marrow derived progenitor cells homing to the sites of injury *in vivo* is expected to be negligible in our *ex-vivo* perfusion system.

Given the rapidity of Cx43 overexpression following rabbit grafting we propose that Cx43 is overexpressed in response to the early surgical stress, rather than flow per se. In line with this hypothesis, cFOS and cJUN, which play a key role in cell proliferation [36], cluster with the Cx43 gene GJA1 following rabbit grafting. Indeed, previous reports demonstrated that cJUN or cFOS control Cx43 promoter activity through a functional AP1 site [37, 38], hence those factors may control Cx43 in response to grafting. On the other hand Cx43 overexpression is partially prevented by external support, implying at least a partial regulation of Cx43 expression by hemodynamic sensitive pathways.

Although the exact mechanisms whereby arterial engraftment leads to Cx43 upregulation remain to be fully unraveled, we observed a strong correlation between the amount of IH and the levels of Cx43, suggesting a role of Cx43 in neointima formation. Conflicting data exist regarding the role of Cx43 in VSMC proliferation and IH formation [2, 5, 7]. Our study clearly supports the concept that Cx43 stimulates VSMC proliferation [2, 7]. Furthermore, both general inhibition of intercellular communication using carbenoxolone or specific blockade of Cx43-made gap junctions prevented myointimal proliferation and the development of IH in human vein grafts, suggesting that Cx43-mediated intercellular communication plays a key role in IH. The development of IH also involves VSMC migration from the media layer to the neointima region. Cx43 knock-down did not impact VSMC migration in our experimental model of wound healing, in contrast with studies showing that Cx43 participates in VSMC transmigration when stimulated with angiotensin II [7] or PDGF [2]. This discrepancy is probably due to the differences between experimental designs and wound healing assay versus transmigration assay. Studies performed in other cell types suggest an opposite role of Cx43 in cell migration [39–41], suggesting a very complex role of Cx43, which clearly depends on the model and cell type studied and warrants further pre-clinical studies before using Cx43-targeted peptides in the treatment of pathologies.

Endothelial cells (EC) are highly sensitive to hemodynamic flow and the frictional forces between blood and the endothelium [42]. Notably, the production of nitric oxide (NO) by EC is a key factor in maintaining quiescent and contractile VSMC [43]. Our data indicate that exposure to arterial hemodynamic rapidly decreases the expression of the major endothelial connexins Cx37 and Cx40, both *in-vivo* and *ex-vivo*. Cx40 [24, 25] and Cx37 [44] are required for proper eNOS expression and function. In line with these data, we previously demonstrated that the expression of the endothelial NO synthase (eNOS) is strongly reduced upon arterial implantation in the EVPS [16]. Thus, despite continuous endothelial coverage as demonstrated by vWf immunostaining, it is likely that the vein graft endothelium is not fully functional when placed in an arterial environment, as suggested by a previous report [45]. In line with this hypothesis, a polymorphism in the Cx37 gene [46], as well as the loss of Cx37 (Cx37-/- mice) [47] increase the risk of atherosclerosis, which share molecular events with IH, including endothelial dysfunction and VSMC proliferation. Unfortunately external reinforcement using the mesh barely improved the expression of these endothelial markers. The combined loss of eNOS, endothelial Cx37 and Cx40, as well as other key endothelial genes likely precludes proper NO production and facilitates IH development. External meshing did not restore the expression of EC connexins Cx37 and 40. In sharp contrast, Cx43 levels, SMCs proliferation and neointima formation were all severely blunted by the external scaffold, which we believe is a viable approach to reduce vein graft failure.

This study highlights Cx43 as an early molecular marker of IH and demonstrates that the development of pathological IH in human vein grafts requires Cx43. Further studies are required to identify the signals passing through gap junctions mediating the increased VSMC proliferation and we cannot exclude that hemi-channels also play a role in this process. Cx43 is mostly expressed by VSMC. Thus, the development of Cx43-targeted pharmacological tools provides an opportunity to manipulate VSMC proliferation while sparing EC connexins. Especially, topical delivery of mimetic peptides blocking Cx43 might provide an exciting strategy in the prevention of IH.

## Supporting Information

S1 Fig The analysis of 57 probe sets demonstrates changes in expression over time after rabbit vein graft. (TIF)

S1 Table List of Primer sequences. (DOCX)

## Abbreviations

VSMC: vascular smooth muscle cells
EC: endothelial cells
Cx: connexin
GJ: gap junction protein
IH: intimal hyperplasia
VGEL: Van Gieson elastic lamina
RNAi: RNA interference
EVPS: ex vivo perfusion system
