# Supplementary figures and images for: Connexin43 Inhibition Prevents Human Vein Grafts Intimal Hyperplasia
Source: PLoS One. 2015 Sep 23;10(9):e0138847. doi: 10.1371/journal.pone.0138847 (PMC4580578; doi:10.1371/journal.pone.0138847)

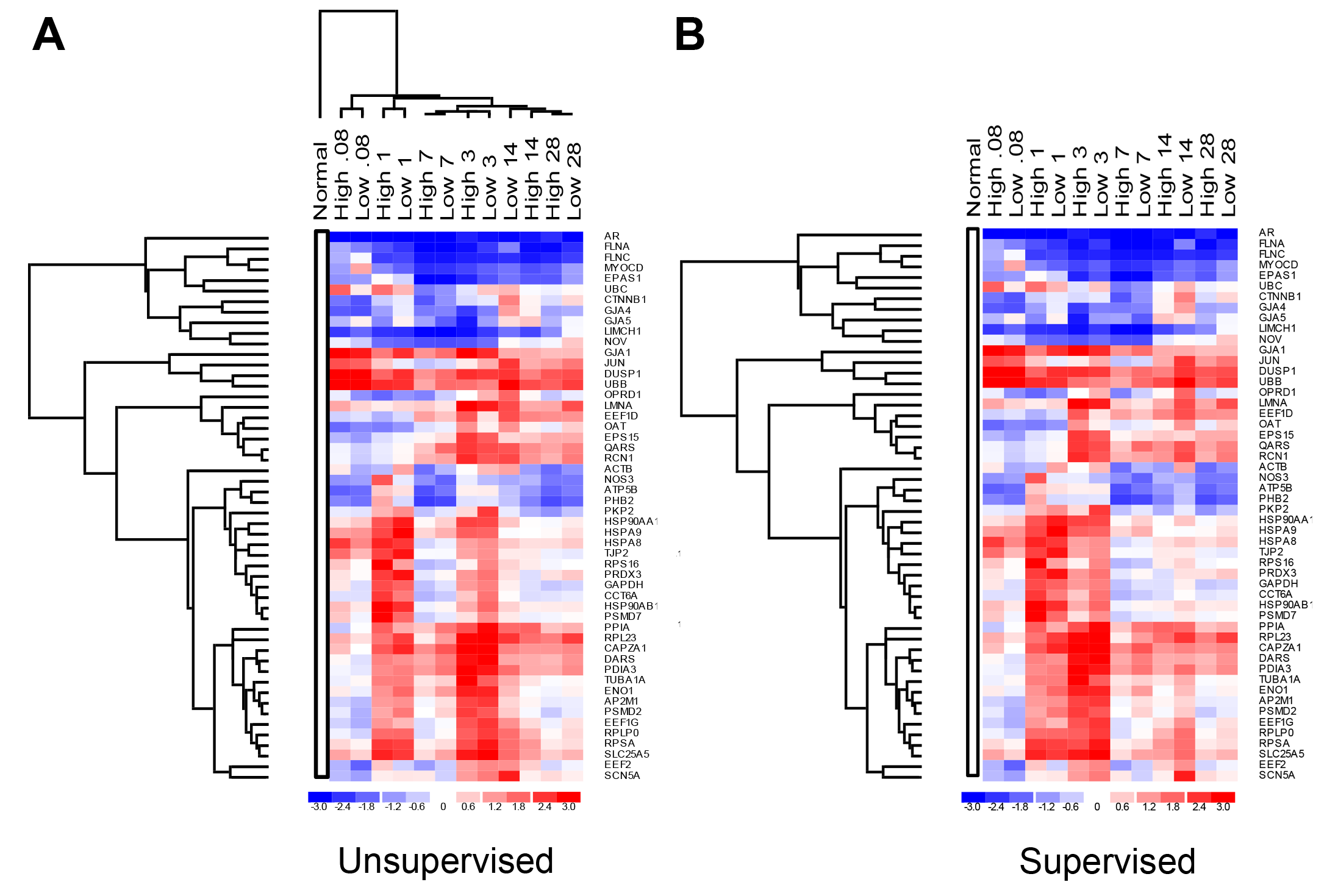

Supplement: S1 Fig — (TIF) [file pone.0138847.s001.tif]
