# Supplementary material for: Connexin43 Inhibition Prevents Human Vein Grafts Intimal Hyperplasia
Source: PLoS One. 2015 Sep 23;10(9):e0138847. doi: 10.1371/journal.pone.0138847 (PMC4580578; doi:10.1371/journal.pone.0138847)
Supplement: S1 Table — (DOCX) [file pone.0138847.s002.docx]

**S1 Table.** Human primers used for the quantitative reverse transcription polymerase chain reaction (RT-PCR).

| ***Gene*** | ***Sense primer (5'-3')*** | ***Antisense primer (5'-3')*** |
| --- | --- | --- |
| **Cx43** | GAACTCAAGGTTGCCCAAAC | TTAGAGATGGTGCTTCCCG |
| **Cx40** | TGGAGGTGGGCTTCATTGTG | TACTTGCTCGGTGACCAGGTTG |
| **Cx37** | ACGAGCAGTCAGATTTCG | GGATGAGAGCCCATTGTAG |
| **GAPDH** | AACTTTGGTATCGTGGAAGG | CAGTAGAGGCAGGGATGATGT |

**S1 Figure: The analysis of 57 probe sets demonstrates changes in expression over time after rabbit vein graft**

**A-B)** Unsupervised (**A**) and supervised (**B**) hierarchal clustering analysis of the 57 time-dependent probe sets (false discovery rate = .05) reveals two distinct clusters with differential expression patterns. Mean expression data at each time point and flow condition are presented across the x-axis, with red representing upregulation and blue representing downregulation compared to normal vein.
